# Structural and Functional Characterization of the FGF Signaling Pathway in Regeneration of the Polychaete Worm *Alitta virens* (Annelida, Errantia)

**DOI:** 10.3390/genes12060788

**Published:** 2021-05-21

**Authors:** Alexandra Y. Shalaeva, Roman P. Kostyuchenko, Vitaly V. Kozin

**Affiliations:** Department of Embryology, St. Petersburg State University, Universitetskaya nab. 7–9, 199034 St. Petersburg, Russia; st041951@spbu.ru (A.Y.S.); r.kostyuchenko@spbu.ru (R.P.K.)

**Keywords:** invertebrates, annelids, *Nereis*, dedifferentiation, blastema induction, evolution, segmentation, axis elongation, SU5402, fibroblast growth factor

## Abstract

Epimorphic regeneration of lost body segments is a widespread phenomenon across annelids. However, the molecular inducers of the cell sources for this reparative morphogenesis have not been identified. In this study, we focused on the role of fibroblast growth factor (FGF) signaling in the posterior regeneration of *Alitta virens*. For the first time, we showed an early activation of FGF ligands and receptor expression in an annelid regenerating after amputation. The expression patterns indicate that the entire regenerative bud is competent to FGFs, whose activity precedes the initiation of cell proliferation. The critical requirement of FGF signaling, especially at early stages, is also supported by inhibitor treatments followed by proliferation assay, demonstrating that induction of blastemal cells depends on FGFs. Our results show that FGF signaling pathway is a key player in regenerative response, while the FGF-positive wound epithelium, ventral nerve cord and some mesodermal cells around the gut could be the inducing tissues. This mechanism resembles reparative regeneration of vertebrate appendages suggesting such a response to the injury may be ancestral for all bilaterians.

## 1. Introduction

Annelids are known for their exceptional regenerative abilities. Their potential to restore considerable body parts, derived from all germ layers, outmatches other classical regeneration models. However, the mechanisms, which allow this process to be completed so efficiently are not described in detail. One of the best developed models of segment recovery are nereid polychaetes, such as *Alitta* (*Nereis*), *Platynereis* and *Perinereis*. Their regeneration is predominantly epimorphic, with some substantial signs of morphallaxis [1,2].

In juvenile *Alitta virens*, following the amputation of the posterior fragment of the body, several segments are restored in about a week, after which anamorphic growth (the addition of segments from the growth zone) continues. Wound epithelium is formed by the fused superficial and intestinal epithelia on the first day post-amputation (dpa) [3]. Along with wound healing, mesodermal cells appear under the wound epithelium, giving rise to the future blastema [4]. Multiple nerve fibers project to the wound area from the severed end of the ventral nerve cord (VNC). During the second dpa, blastemal and epithelial cells exit from the cell cycle arrest and proliferate. By three dpa, the patterning of the regenerative bud leads to segregation of three areas: pygidium, growth zone, and material of future segments [3]. On the fifth dpa, an external groove appears on the posterior border of the newly formed segment, which already contains a nerve ganglion, a pair of coeloms, and transverse and longitudinal muscles. Expression of tissue-specific marker genes indicates the succession of germ layers during regeneration in nereids and the major role of local cell dedifferentiation [4,5,6]. The only known inductive interactions involved in late regeneration steps are Wnt signals [7].

In other animals, one of the key regulators of the reparative processes is fibroblast growth factors (FGF) [8,9,10,11,12]. These molecules are widespread in eumetazoan phyla, demonstrating a significant divergence in primary sequence and number of paralogs [13,14,15,16]. FGFs bind to tyrosine-kinase receptors (FGFRs) and activate multiple intracellular pathways, such as MAP-kinase, PI3-Akt, and PLCγ. These pathways regulate different cellular responses: proliferation, differentiation, migration, epithelial-mesenchymal transition, suppression of apoptosis and inflammation [17,18,19]. Such a pleiotropic effect is achieved by a different cellular context and various paralogous genes. Mammals have 22 different ligands grouped into 7 subfamilies. Detailed classification and functional analysis of paralogous molecules are done for other vertebrates, too [14,15,16,20,21,22,23,24,25]. On the contrary, an unambiguous affiliation of the identified FGF members in invertebrates remains problematic [9,15,18,26,27,28]. A detailed molecular and functional evolution of FGFs in the context of regeneration is yet to be fully elucidated.

Vertebrate models of limb, tail and fin regeneration demonstrate that FGF molecules mediate the signal to initiate the formation of the blastema and subsequent proliferation of its cells [8,10,28]. The role of the FGF pathway is best described in axolotl limb regeneration. The wound epithelium of the amputated limb becomes the source of FGFs 1, 2 and 8, which are received by underlying blastemal cells, and cause a mitogenic effect. Blastemal cells subsequently become the source of FGF10, which influences epithelial cells, establishing a positive feedback loop [29,30]. Another remarkable role of FGFs was shown on the accessory limb model. Early studies have demonstrated that nerve deviation to the wound site leads to forming blastema instead of wound healing, which normally occurs [31]. However, recent studies show that an FGF-soaked bead can substitute the deviated nerve and result in limb or tail development [32,33]. A study performed on the zebrafish fin regeneration has also demonstrated that FGF signaling is essential and employs a similar activation mechanism. Wound epithelium induces blastema by FGF20a. However, in this case, primary FGF signals do not trigger proliferation but induce underlying mesenchymal cells to participate in blastema formation. Further blastemal cells become the source of FGF3/10a, which supports proliferation [8].

After identifying the first growth factor in mammals in 1973, it took almost 20 years to identify a substance with similar effects in invertebrates. Noteworthy, it was made on the nereid polychaete *Nereis* [34]. Based on immunological properties and biological activity, the nereid growth factor was homologized to a mammal FGF, but the amino acid sequence and the definite phylogenetic position were not determined. In the context of invertebrate regeneration, FGF activity is described for cnidarians [9,35,36], planarians [37,38] and a brittle star [12]. These works demonstrate that FGFs have a specific expression and play distinct roles in these organisms.

The variety of ligands and receptors of the FGF pathway has never been described in detail for any spiralian (lophotrochozoan) animal. The expression of the components of the FGF pathway is shown in embryonic and larval development of brachiopods [39,40], a phoronid [40] and, partially, in an annelid *Capitella teleta* [41].

In this study, we focused on the role of FGF signaling in the posterior regeneration of *Alitta virens* (formerly *Nereis virens*). Our work elucidates the molecular evolution, expression patterns, and functional requirement of the protostome FGFs. These findings are important for understanding when and how FGFs acquired their incredible capacity to promote regeneration across the whole bilaterian clade.

## 2. Materials and Methods

### 2.1. Animals

Spawning epitoke individuals of *A. virens* were caught in summer near the Marine Biological Station of SPbSU at the White Sea. Laboratory culture of embryos was obtained by artificial fertilization [42]. Animals grew for 2–3 months in small aquariums with natural or artificial seawater. The posterior third of the juveniles’ body was amputated, and then the animals were left to regenerate for various periods at +18 °C. When the animals reached the preferred stage, they were anesthetized with 7.5% MgCl_2_ mixed with artificial seawater (1:1) and fixed in 4% PFA on 1.75x PBS with 0.1% Tween-20. The samples were left at +4 °C overnight and then either rinsed with phosphate buffer without sodium azide for EdU detection or put in 100% MetOH for longer storage at 20 °C.

### 2.2. SU5402 and U0126 Treatments

To inhibit FGF signaling, the regenerating juveniles were treated with 50 µM SU5402 (Sigma-Aldrich) or 40 µM U0126 (Promega). Inhibitors were diluted with DMSO (stock concentration U0126–10 mM, SU5402–25 mM), aliquoted, and stored for no more than 2.5 months at −20 °C. The inhibitors were added to artificial seawater in which regenerating worms were kept. For prolonged incubation, the solution was changed every other day. The controls were incubated in 0.4% DMSO.

### 2.3. EdU Labeling and Fluorescent Stainings

Proliferating cells were labeled by incubating the worms for 15 min with 5 µM EdU (5-ethynyl-2′-deoxyuridine, analogous to thymidine) just before anesthetization and fixation in PFA. After washes with PBS and one fast wash with 0.1 M Tris buffer (pH = 8.5), the samples were put in the click-reaction mixture for 45 min at room temperature. The mixture consisted of 100 mM Tris (pH = 8.5), 4 mM CuSO_4_, 2 µM sulfo-cyanine-5-azide (Lumiprobe), and 50 mM ascorbic acid in deionized water (I. Borisenko, personal communication). After this, the samples were rinsed with PBT and subjected to immunocytochemical detection of acetylated tubulin and nuclei labeling with DAPI. Acetylated tubulin was detected using primary antibodies (Sigma T6793, dilution 1:250) and fluorescent secondary antibody anti-mouse AF568 (Invitrogen, dilution 1:500) as previously described [3]. Incubation in antibodies took 1–2 days at +4 °C. After this, the samples were left in DAPI (1 µg/mL) for 1–2 h at room temperature or +4 °C overnight, then rinsed with PTW and stored in 90% glycerol.

F-actin labeling was performed independently of the other types of detection using phalloidin-AF488 (Invitrogen A12379, concentration 5 units/mL) as previously described [3]. The samples were incubated for 2 h at room temperature, then rinsed with phosphate buffer and stored in 90% glycerol.

### 2.4. Sequence Retrieval and Phylogenetic Analysis

Sequences of FGF and FGFR were found in an unannotated transcriptome database for *Alitta virens* (local resource), *Platynereis dumerilii* databases (http://4dx.embl.de/platy; http://pdumbase.gdcb.iastate.edu/platynereis/controller.php?action=blast accessed on 1 November 2018), genome assembly for *Praesagittifera naikaiensis* [43] (https://marinegenomics.oist.jp/p_naikaiensis/viewer?project_id=71 accessed on 1 April 2021), *Capitella teleta* and *Lottia gigantea* [44] (https://genome.jgi.doe.gov/portal/ accessed on 1 April 2021) and in the publicly available molecular genetic databases GenBank and UniProt via TBLASTN search. The sequences were analyzed and aligned in the CLC Workbench. The amino acid alignments (Supplementary Materials Data S6 and S7) were used for further phylogenetic analysis. The consensus tree was inferred using MrBayes3.7.2a on the CIPRES portal (https://www.phylo.org/ accessed on 1 April 2021). Two runs of 1,000,000 generations each with every 100 generation sampling were performed on 4 chains with a burn-in fraction of 0.25. In addition, maximum-likelihood analysis was performed using the IQtree online server (http://iqtree.cibiv.univie.ac.at/ accessed on 1 April 2021), models of amino acid substitution were selected automatically based on the input data, bootstrap = 1000. Domain organization of the sequences was established using the online programs PROSITE (https://prosite.expasy.org/ accessed on 1 April 2021) and SMART (http://smart.embl-heidelberg.de/ accessed on 1 April 2021).

### 2.5. Cloning of the cDNA

For RNA probes for in situ hybridization, the fragments of four candidate sequences were cloned. We amplified the fragments from 1.1 to 2.4 kb, which contained either partial or full coding sequences, using cDNA of regenerating *A. virens* as a template and Phusion® high-fidelity (NEB) or Encyclo (Evrogen) DNA polymerase. Primer design was made using Primer3 (http://primer3.ut.ee/ accessed on 1 March 2019). Gene-specific primers were made for each gene of interest *Avi-fgfr2* forward primer CTGGGGACACCCATCAGTTG, *Avi-fgfr2* reverse primer CCCAAGCCATATCCCCCTAC (PCR program: 98°C–2′ → (98 °C–15″ → 66 °C–45″ → 72 °C–1′30″) × 9 → (98 °C–15″ → 64 °C–45″ → 72 °C–1′30″) × 35 → 72 °C–5′.); *Avi-fgfr1* forward primer AACGGTGTTGTCAGGGTCGG, *Avi-fgfr1* reverse primer GCTTCCAACGCTGCTAACCG (98 °C–2′ → (98 °C–15″ → 62 °C–20″ → 72 °C–30”) × 35 → 72 °C–5′.); *Avi-fgfA* forward primer CCACCAGTTTCAACACCGCG, *Avi-fgfA* reverse primer AGTCCTCCCTTCTTTCGCCG (95 °C–3′ → (95 °C–30″ → 62 °C–45″ → 72°C–1′) × 35 → 72 °C–5′.); *Avi-fgf8/17/18* forward primer AGACTTCTCCAGCTCTGCGG, *Avi-fgf8/17/18* reverse primer ACAATGCGCCTCCTTTTCCC (95 °C–3′ → (95 °C–30″ → 63 °C–45″ → 72 °C–1′) × 35 →72 °C–5′). The PCR products were ligated into pAL2-T (Evrogen TA cloning kit) and used to transform chemically competent *E. coli* (One Shot® TOP10). After colonies with correct insert were obtained and checked by sequencing, we synthesized the digoxigenin-labeled RNA probes used for in situ hybridization.

### 2.6. Whole-Mount In Situ Hybridization

The protocol was performed as described previously [4]. The samples were rehydrated from MetOH, then rinsed in PTW, treated with proteinase K (100 µg/mL) for 2.5–3 min at +22 °C, twice rinsed in glycine (2 mg/mL), postfixed with 4% PFA on PTW for 20 min, and washed in PTW before pre-hybridization. After overnight incubation with the probe and subsequent washes, the samples were blocked in 5% sheep serum, followed by anti-digoxigenin AP antibodies overnight incubation (dilution 1:2000). The staining was performed with NBT/BCIP, followed by washing in PTW and mounting in 90% glycerol.

The stained samples were also embedded in glycolmethacrylate [45] and sectioned in series of 5 µm sections.

### 2.7. Data Visualization

Confocal images were obtained with Leica TCS SPE confocal microscope. In situ hybridization results were visualized using DIC optics with Axio Imager D1 microscope (Carl Zeiss). The optical sections were combined in stacks and converted to video files (Supplementary Materials Data S2–S5) by ImageJ. Schemes were made in Adobe Illustrator.

### 2.8. Western Blotting

Amputated animals were incubated in inhibitors or DMSO from 0 to 2 dpa as described above. The posterior-most 15 segments and the regenerative bud were cutoff and homogenized in lysis buffer, containing Tris 50 mM (pH = 7.4), SDS 1%, NaCl 150 mM, EDTA 10 mM, PMSF (1:100) and a phosphatase inhibitor (100 mM Na_3_VO_4_, 250 mM NaF, 50 mM sodium glycerophosphate, 50 mM sodium pyrophosphate, dilution 1:50). Then the samples were centrifuged at +4 °C for 10 min at 14,000 *g*. An aliquot of fresh supernatant was used for measuring protein concentration by the Bradford method with BSA as a standard. The supernatant was mixed with loading buffer (pH = 6.8), denatured for 10 min at 95 °C and cooled at room temperature. The samples were loaded in 10% polyacrylamide gel (50 µg of protein on each lane), resolved with SDS–PAGE under reducing conditions, and transferred to the PVDF membrane (Amersham) by electroblotting. To control the blotting results, the membrane was stained with Ponceau S for 30 s, rinsed with water and blocked in 5% sheep serum and 1% BSA diluted in TBST for 1 h. Then the membrane was incubated in the primary rabbit antibodies against phospho-p44/42 MAPK (Erk1/2) (Thr202/Tyr204) (Cell signaling technology #4370, dilution 1:250) overnight at +4 °C. After several washes in TBST incubation in the secondary antibodies anti-rabbit HRP (Thermo #A16096, dilution 1:1000) was performed for 2 h at room temperature. All membrane incubations and washes were performed using an orbital shaker. After the membrane was rinsed with TBST 3 times for 10 min and once with TBS (pH = 7.2), it was submerged into developing solution pH = 7.0 (DAB 0.5 mg/mL, 0.1 M imidazole, 0.01% H_2_O_2_) for 30 min. Then the membrane was rinsed with water and photographed.

## 3. Results

### 3.1. Sequence Analysis

To characterize the FGF signaling pathway components in *A. virens*, we searched the local databases for candidate sequences, followed by the domain mapping and phylogenetic analysis. Two candidate FGF genes (*Avi-fgf8/17/18* and *Avi-fgfA*) and two genes encoding FGFR (*Avi-fgfr1* and *Avi-fgfr2*) were identified. The core part of the ligands’ sequence is rather small, approximately 120aa, and highly variable [13,14,16]. For this part, the similarity of *A. virens* FGFs with the sequences of other animals was moderate (the proportion of identical amino acid residues did not exceed 23%). The core FGF sequences of *A. virens* and *P. dumerilii* were more similar with each other, 65% and 41% for FGF8/17/18 and FGFA, respectively.

**Figure 1:**
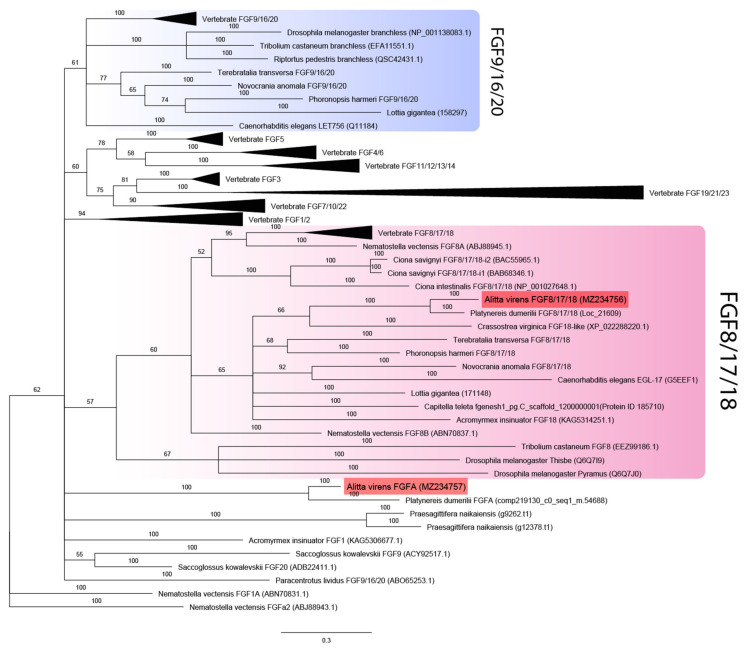
FGF ligands phylogeny (Bayesian inference). *A. virens* sequences *Avi-FGF8/17/18* and *Avi-FgfA* are marked in red. Branch labels demonstrate probability (percent). Polytomy indicates the branch support less than 50%. Collapsed branches are in black triangles. The scale bar shows the number of amino acid substitutions per site.

Additional bona fide FGF candidates were looked for in genome datasets for spiralians *Capitella teleta* and *Lottia gigantea* [44] and the basally divergent bilaterian *Praesagittifera naikaiensis* [43]. Two unique sequences with characteristic domain organization were identified in the acoel worm *P. naikaiensis*, one in the polychaete *Capitella teleta*, and two in the mollusk *L. gigantea*. Phylogenetic analysis showed that all eumetazoans tested (except for *P. naikaiensis*) possessed members of the FGF8/17/18 subfamily (Figure 1). One of the two candidates in nereids and *L. gigantea*, as well as a single predicted gene from the *C. teleta* genome, was reliably assigned to the FGF8/17/18 subfamily. These sequences cluster with previously analyzed *FGF8* orthologs of ecdysozoans, lophophorates, and chordates. The second paralog of the FGF ligand of *A. virens* and *P. dumerilii* and both candidates of the acoel *P. naikaiensis* did not cluster with any clade, so their phylogeny is left unresolved. In contrast, the *L. gigantea* FGF candidate formed a clade together with lophophorate *FGF9/16/20* genes. The sister clades are undebatable homologs of *FGF9/16/20* of vertebrates and *C. elegans* and obscure insect-specific *branchless* genes. Our data show that the FGF paralogs *pyramus* and *thisbe*, which were previously suggested to be an independent acquisition of insects, are copies of *FGF8/17/18*, while *branchless* may represent a derived variant of insect *FGF9/16/20*. Precisely these two subfamilies (FGF8/17/18 and FGF9/16/20) are enriched with genes from diverse taxa, which may explain low levels of support for the corresponding clades. Noteworthy, among *FGF8/17/18* genes, the most derived sequences belong to insects, but in the inferred FGF9/16/20 subfamily, the longest branches are formed by the representatives of spiralians (a mollusk and a phoronid). Altogether these results provide a new perspective on the FGF repertoire in protostomes.

**Figure 2:**
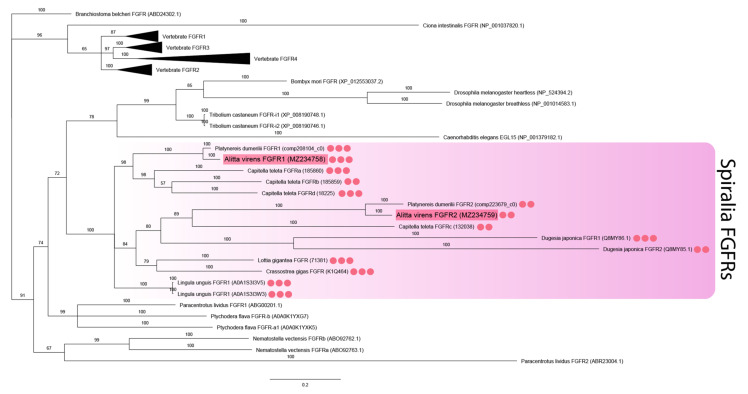
FGF receptors phylogeny (Bayesian inference). *A. virens* sequences *Avi-fgfr1* and *Avi-fgfr2* are marked in red. Red circles represent the number of Ig-like domains in spiralian FGFRs. This parameter for *C. teleta* is inferred according to the previously published analysis [46]. Branch labels demonstrate probability (percent). Polytomy indicates the branch support less than 50%. Collapsed branches are in black triangles. The scale bar shows the number of amino acid substitutions per site.

The analysis of FGF receptors revealed both conserved and lineage-specific characters. Several Ig-like domains (from two to five), a transmembrane domain, and a tyrosine kinase domain are characteristic of metazoan FGF receptors [46]. The receptors’ sequences were aligned by the tyrosine kinase domain since the number of Ig-like domains and their sequences tend to diverge. The two FGF receptor sequences of *A. virens* were found to be closely related to the FGFRs of other spiralians (branch support 100%) (Figure 2). This clade demonstrates lineage-specific diversification events. *Avi-fgfr1* and *Avi-fgfr2* are independent duplicates and do not correspond to the vertebrate FGFR1 and FGFR2 subfamilies. *C. teleta* has four paralogs, which are presumably the result of a more recent expansion. Comparing the number of Ig-like domains in the cluster of spiralian sequences, we showed that their number correlated with the location on the phylogenetic tree. Paralogous sequences of *A. virens* and *P. dumerilii* FGFRs contained either three or two Ig-like domains, while the tyrosine kinase domain showed a significant divergence rate.

These results suggest that the evolution of FGF components in Metazoa was predominantly independent. Both cnidarians and different bilaterian clades acquired lineage-specific FGF subfamilies. FGF8/17/18 seems to be the most enriched and indispensable subfamily. Nevertheless, the set of FGF genes could be replenished or could undergo secondary losses in each taxon. This is indicated by the varying number of paralogs, the potential horizontal transfer of *branchless* [16] and the differences in the number of conserved domains (for example, Ig-like domains in annelid receptors).

### 3.2. Expression Patterns of the FGF Signaling Genes

**Figure 3:**
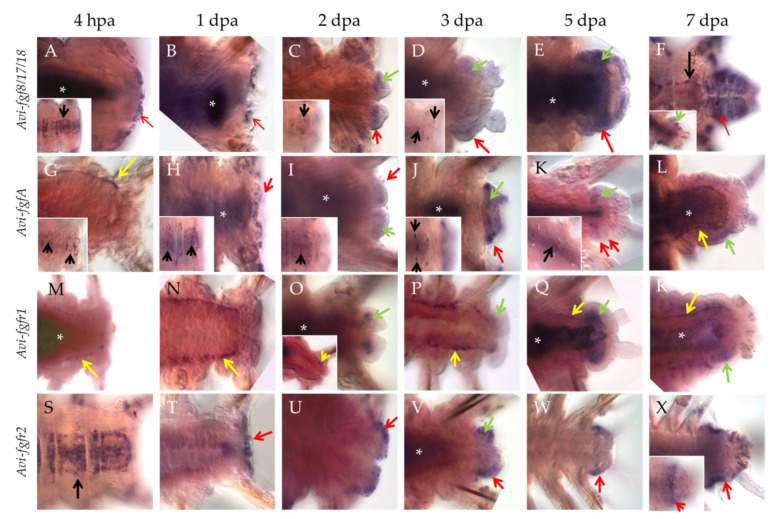
Whole-mount in situ hybridization, which shows mRNA expression of FGF and FGFR genes in posterior regeneration of *A. virens*. (**A**–**F**) *Avi-fgf8/17/18*, (**G**–**L**) *Avi-fgfA*, (**M**–**R**) *Avi-fgfr1*, (**S**–**X**) *Avi-fgfr2.* In all planes, the anterior end is to the left. Red arrows indicate expression signal in the wound epithelium (**A**,**B**,**H**,**T**) or epidermis of the regenerative bud; green arrows, expression signal in the mesoderm-derived blastema; yellow arrows, expression signal in the mesodermal cells around the gut; black arrows, expression in the ventral nerve cord. Asterisks mark nonspecific staining in the gut lumen. Inserts show a different focal plane of the corresponding stage specimen.

Already 4 h after amputation (hpa), the transcripts of *Avi-fgf8/17/18* appear in the cells of the VNC ganglia (Figure 3A, insert), the lateral epidermis of the last segments, as well as in the wound epithelium (Figure 3A, arrow). At later stages, expression in the nervous system becomes less extensive and persists in single cells (Figure 3C,D inserts). By 1 dpa, mRNA expression is observed only within the wound epithelium (Figure 3B, arrow). Towards more advanced stages of regeneration, the signal remains in the superficial epithelium (Figure 3C–F, red arrows) and developing cirri, and also appears in the cells of the blastema (Figure 3C,D, Supplementary Materials Data S2), which retain it in the anterior parts of the regenerative bud (Figure 3E,F, green arrows). By the 7 dpa stage, *Avi-fgf8/17/18* expression encompasses most of the regenerated epidermis, neuroectoderm and the old VNC ganglia (Figure 3F).

Individual ventral cells within the VNC (Figure 3G, insert, black arrows) show *Avi-fgfA* expression as early as 4 hpa. The number of these neural elements increases towards the site of amputation, starting with 4 cells in the first 1–2 setigerous segments and ending with 16 cells in the last 8–10 segment. Expression in the nervous system remains up to 5 dpa (Figure 3H–K inserts). At the stage 1 dpa, we observed an additional ectodermal domain in the wound epithelium (Figure 3H), which at later stages remains in the epithelium of the regenerative bud (Figure 3I–K, red arrows, Supplementary Materials Data S3) and in cirri. Ectodermal expression has a different intensity along the AP axis so that the most intense domains are confined to the middle of each segment anlage, while the weakest ones coincide with segmental boundaries (Figure 3J–L). Furthermore, from the earliest studied time point (4 hpa), we found *Avi-fgfA* expression in mesodermal cells around the gut in the segments near the amputation site (Figure 3G, yellow arrow). These cells can be classified as elements of the coelom wall (myoepitheliocytes) or free coelomocytes. Later on, these *Avi-fgfA*-positive cells are detectable at 3 dpa and 7 dpa (Figure 3L, yellow arrows). This spotted domain merges with the faint staining in blastemal cells, which eventually differentiate in coelomic sacks (Figure 3I–L, green arrows).

*Avi-fgfr1* demonstrates an exclusively mesodermal expression pattern during regeneration. Like the *Avi-fgfA*, the first response to the amputation from *Avi-fgfr1* expression appears at 4 hpa in the individual cells around the gut (Figure 3M). The visual level of staining intensity in this domain gradually increases towards the injury site; the number of cells with a signal is also uneven and tends to increase along the anterior-posterior axis. This expression profile persists to later stages up to 7 dpa (Figure 3M–R, Supplementary Materials Data S4). Histological sections clarified the localization of the mesodermal cells, confined to visceral and parietal coelomic walls (Supplementary Materials Data S1). On the second dpa, at the posterior end of the supposedly migrating *Avi-fgfr1*-positive mesodermal cells of the old segments, an expression domain in the developing blastema becomes apparent (Figure 3O). As the bud grows and differentiates, the expression remains in the mesodermal tissues and is not present in the epithelium (Figure 3O–R).

The expression of *Avi-fgfr2* exhibits a predominantly ectodermal tissue specificity. This gene is active mainly in the epithelium and, at a much lower level, in the mesodermal cells of the regenerate. The first cells with transcripts appear at 4 hpa across the VNC ganglia (Figure 3S). The intensity of the signal gradually fades away in the anterior direction. By 1 dpa, the expression is observed in the wound epithelium (Figure 3T). Later on, the expression remains in the epithelium anterior to the pygidium (Figure 3U–X, Supplementary Materials Data S5). A sharp border of this superficial domain demarcates the growth zone (Figure 3X, insert). At the ventrolateral epidermis of the segmented regenerate (5–7 dpa), *Avi-fgfr2* expression has several bilaterally symmetric stripe-shaped domains with a brighter signal, which coincide with segmental furrows and putative borders (Figure 3X). In addition, a faint signal is detectable in a few blastemal cells at 2–3 dpa (Figure 3V).

### 3.3. Inhibition of FGF signaling by SU5402 and U0126

**Figure 4:**
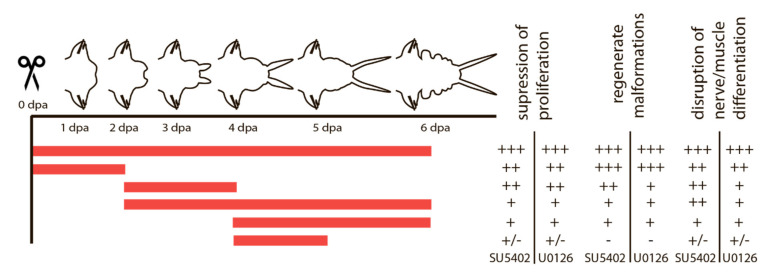
Summary of SU5402 and U0126 treatment and its effect on proliferation, morphogenesis and tissue differentiation. Periods of treatment are marked in red. The outcome varies from a strong effect (+++) to no visible differences (-).

Given the importance of cell proliferation and subsequent differentiation of the proliferated cells at certain stages, we assumed that these processes might be under the control of FGF signaling. This assumption prompted us to experiments with pharmacological inhibitors of the FGF signaling pathway, which affect it at different levels. SU5402 suppresses the interaction between the ligand and the receptor, and U0126 suppresses the MAP kinase pathway, which is also activated by FGF signaling. We used various schemes of treatment with inhibitors, which are summarized in Figure 4.

**Figure 5:**
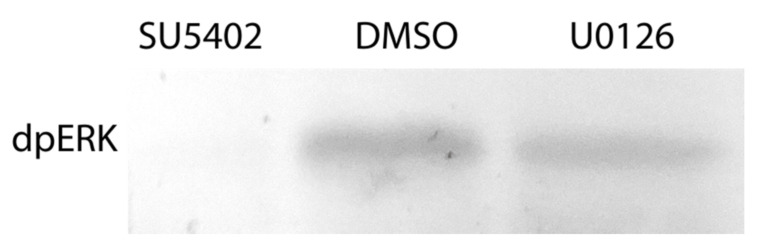
Phosphorylated MAP-kinase detection after inhibition of the FGF pathway. Incubation in DMSO is used as a control. 42 kDa band is present in control DMSO but not in SU5402-treated regenerates. A decreased intensity in the U0126-treated sample indicates a partial MAPK downregulation, which nevertheless has an apparent stage-specific phenotypic expression (Figure 4, Figure 6, Figure 7, Figure 8 and Figure 9). A similar amount of transferred protein was controlled by Ponceau S staining (not shown).

The effects of the inhibitors on regenerating animals were investigated using morphological, immunofluorescent, and Western blot analysis. Antibodies against phosphorylated MAP-kinase (dpErk1/2) previously tested on *A. virens* [47] revealed in the control sample a single band of 42 kDa (Figure 5), which corresponds to the expected Erk molecular mass. This indicates an active state of MAPK cascade during regeneration, which is downregulated by SU5402 and U0126 (Figure 5). Since MAPK is a conserved target of FGF signaling and the similar overall effect of the two inhibitors is apparent (Figure 4), we confirm the specificity of the used pharmacological agents.

#### 3.3.1. Suppression of FGF Signaling Immediately after Amputation

**Figure 6:**
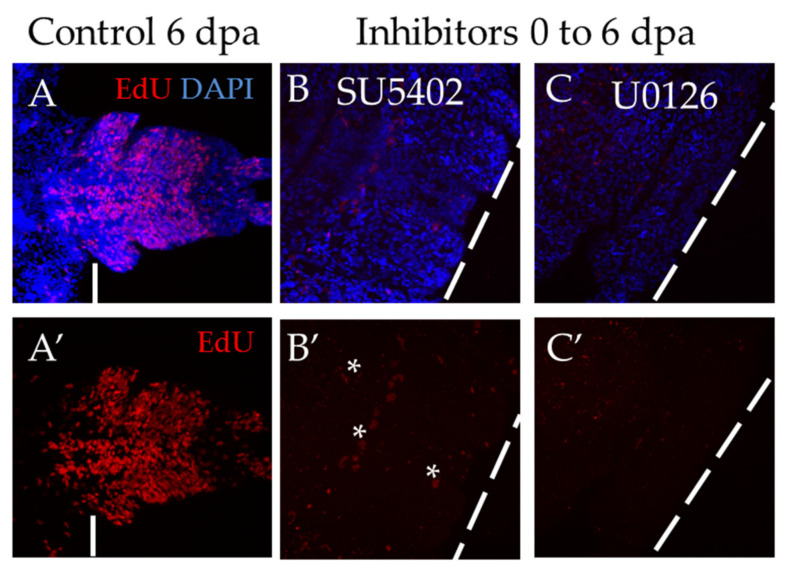
Suppression of proliferation during long-term incubation in SU5402 and U0126, starting from the moment of amputation. All planes are maximum projections of confocal Z-stack of ventral views (anterior is to the left) of posterior regeneration of *A. virens*. (**A**–**C**) EdU labeling in red, nuclear staining with DAPI in blue. (**A′**–**C′**) EdU in red. The dotted line indicates the level of amputation. Asterisks, nonspecific autofluorescence of the exocrine glands. (**A**,**A′**) control with DMSO at 6 dpa, (**B**,**B′**), (**C**,**C′**) after 6 days of incubation in SU5402 (**B**,**B′**) or U0126 (**C**,**C′**). In comparison with control, there is no regenerative bud at the stage of 6 dpa (**B**,**C**), while EdU labeling shows an almost complete suppression of proliferation (**B′**,**C′**).

The most severe effect was observed when the animals were exposed to inhibitors for 6 days, starting immediately from the moment of amputation (Figure 6). Both inhibitor treatments showed a complete suppression of proliferation, which resulted in the absence of the blastema and the regenerative bud. The standard EdU incorporation experiment recovered no more than a dozen weakly labeled cells (Figure 6B′,C′), while normally, at that stage, there are about several thousand EdU+ cells (Figure 6A′). We also found that if the animals are washed out from the solution of inhibitors and cultured under normal conditions, the ability to regenerate is fully restored.

#### 3.3.2. Suppression of FGF Signaling from the Moment of Amputation up to 2 dpa

**Figure 7:**
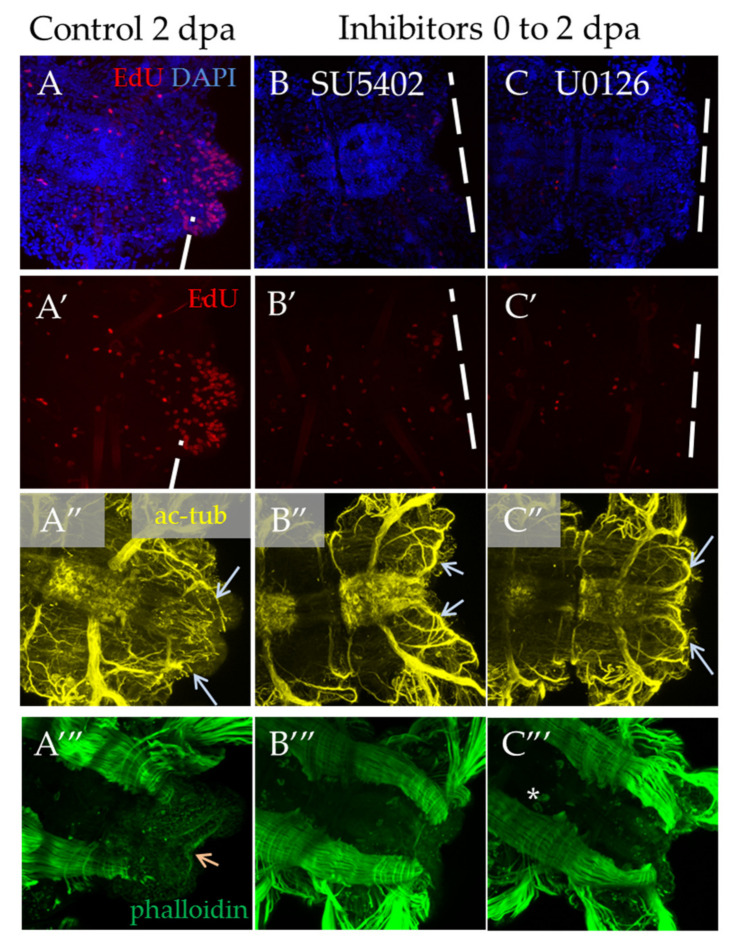
Proliferation and differentiation of nerves and muscles after inhibition of FGF pathway starting from amputation to 2 dpa. All planes are maximum projections of confocal Z-stack of ventral views (anterior is to the left) of posterior regeneration of *A. virens*. (**A**–**A‴**) control in DMSO, (**B**–**B‴**) SU5402, (**C**–**C‴**) U0126. (**A**–**C**) EdU labeling in red, nuclear staining with DAPI in blue; (**A′**–**C′**) EdU labeling in red; (**A″**–**C″**) antibody labeling against acetylated tubulin (yellow) shows the nervous system, and gray arrows indicate lateral nerves, which at this stage grow into the regenerative bud. (**A‴**–**C‴**) phalloidin staining of the muscular system, an arrow indicates pygidial circular muscles (**A‴**), which are absent in (**B‴**,**C‴**). The asterisk in (**C‴**) is the autofluorescence of the exocrine glands.

In the case of a shorter inhibition of FGF signaling, starting from the moment of amputation to 2 dpa, we observed a significant suppression of proliferation and no signs of blastema development or any outgrowth (Figure 7B′,C′). Normally, an early blastema is already formed at this stage [3]. The observed disturbances in the nervous and muscular systems are associated with the absence of the regenerative bud. As regards neuromorphology, at this stage, we noted neurites from the lateral nerves projecting through the wound epithelium (Figure 7B″,C″, arrows), but no innervation from the VNC posterior end characteristic of control 2 dpa regenerates. In the muscular system (Figure 7A‴–C‴), which at this stage is characterized by forming circular pygidial muscles, this structure was not revealed in the treated samples.

#### 3.3.3. Suppression of FGF Signaling from 2 to 4 dpa and from 4 to 5 dpa

**Figure 8:**
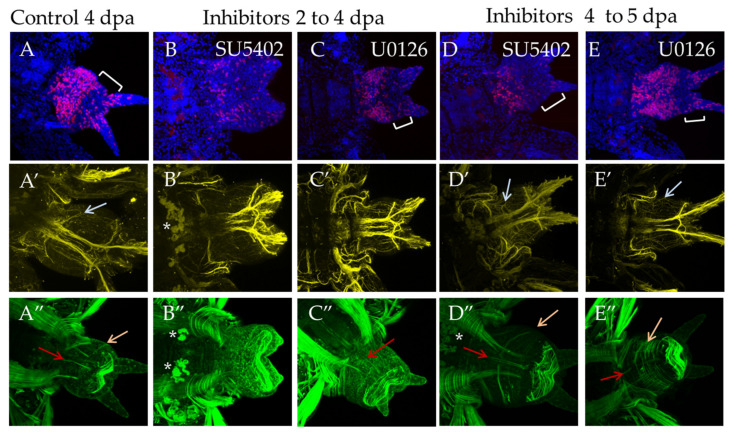
FGF inhibitors influence proliferation and differentiation of muscles and nerves in experiments starting from 2 dpa to 4 dpa ((**B**–**B″**) and (**C**–**C″**) in SU5402 or U1026, respectively) or from 4 dpa to 5 dpa ((**D**–**D″**) and (**E**–**E″**) in SU5402 or U1026, respectively). All planes are maximum projections of confocal Z-stack of ventral views (anterior is to the left) of posterior regeneration of *A. virens*. Planes (**A**–**A″**) DMSO control at 4 dpa stage. (**A**–**C**) EdU labeling in red, nuclear staining with DAPI in blue, bracket indicates pygidium region (in B there are no cirri and no apparent pygidium region); (**A′**–**C′**) antibody labeling against acetylated tubulin (yellow) shows the nervous system, and gray arrows indicate thin parapodial nerves (absent in (**B′**,**C′**)). (**A″**–**C″**) phalloidin staining of the muscular system, red arrows indicate ventral median longitudinal muscle and orange arrows indicate oblique muscles in each formed segment (**A″**,**D″**,**E″**). Asterisks, autofluorescence of the exocrine glands.

At stage 4 dpa, a new segment is formed in control animals, and differentiation of its metameric elements within the nervous and the muscular system occurs. The pygidium region becomes more prominent (Figure 8A, bracket). During incubation from 2 dpa to 4 dpa, suppression of proliferation was noted in comparison with the DMSO control, the effect being more pronounced for SU5402 (Figure 8B). The disturbed morphology of the bud resembles a less advanced stage of the regenerative process (Figure 8B,C). Subtle differences were observed in the nervous and the muscular system. Normally, at this stage, the first pair of parapodial nerves is restored (Figure 8A′, arrow), but the inhibitors blocked their formation completely (Figure 8B′,C′). SU5402 had a more prominent effect on the differentiation of the muscular system. Despite forming the circular muscles of pygidium when both inhibitors were used, incubation in SU5402 did not result in the recovery of longitudinal muscles (Figure 8B″), which were observed in U1026 (Figure 8C″, orange arrow).

Incubation in inhibitors for 24 h starting from the stage 4 dpa had a less pronounced effect on cell proliferation and morphology of the regenerate (Figure 8D,E). The nervous and the muscular system are almost unaffected and show an almost normal differentiation of the parapodial nerves and circular, longitudinal and oblique muscles (Figure 8D′,E′,D″,E″, arrows).

#### 3.3.4. Suppression of FGF Signaling from 2 or 4 to 6 dpa

**Figure 9:**
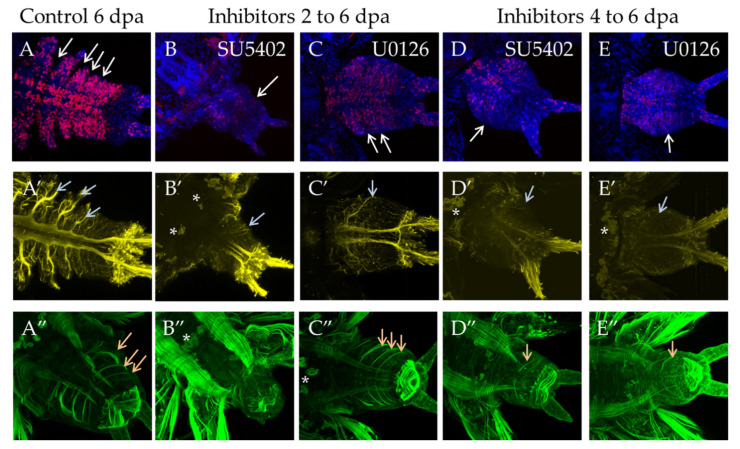
FGF inhibitors influence proliferation and differentiation of muscles and nerves in experiments starting from 2 dpa to 6 dpa (planes (**B**–**B″**) and (**C**–**C″**) in SU5402 or U1026, respectively) or from 4 dpa to 6 dpa (planes (**D**–**D″**) and (**E**–**E″**) in SU5402 or U1026, respectively). All planes are maximum projections of confocal Z-stack of ventral views (anterior is to the left) of posterior regeneration of *A. virens*. Planes (**A**–**A″**) DMSO control at 6 dpa stage. (**A**–**C**) EdU labeling in red, nuclear staining with DAPI in blue, arrows point to separated segment borders; (**A′**–**C′**)—antibody labeling against acetylated tubulin (yellow) shows the nervous system, gray arrows indicate lateral nerves. (**A″**–**C″**) phalloidin staining of the muscular system, arrows indicate oblique muscles in each formed segment (**A″**,**C″**,**D″**,**E″**). Number of these muscles varies from 0 (**B″**) to 1 (**D″**,**E″**) or 3 (**A″**,**C″**). Asterisks, autofluorescence of the exocrine glands.

Incubation in inhibitors from the 2 dpa to 6 dpa stage had a more prominent effect in SU5402. Morphologically the regenerative bud does not correspond to 6 dpa control, since the proper number of segments did not form (Figure 9B, white arrow) and the distinctive structures of the nervous system, such as thick parapodial nerves, did not form, either, with only a thin network of nerves forming instead (Figure 9B′, gray arrow). In the abnormal muscular system, the circular muscles of the pygidium are weakly developed, and oblique muscles are not developed at all (Figure 9B″). In U0126, morphological differences from the normal development were less apparent as many as usual intersegmental furrows were formed (Figure 9C, white arrows). Parapodial nerves were formed in the nervous system (Figure 9C′, gray arrow). The newly formed oblique and longitudinal muscles exactly correspond to the 6 dpa stage (Figure 9C″, orange arrows).

During 48 h incubation starting from 4 dpa, the effect was also noticeable. Although the formation of the first new segment was not obvious to the eye, confocal reconstructions showed its anlage (Figure 9D,E, white arrows) and the formation of its neural and muscular structures. The parapodial nerves were rather thin (Figure 9D′,E′, gray arrows). The longitudinal and the oblique muscles of the future segment were differentiated (Figure 9D″,E″, orange arrows). During this time period, the regeneration slowed down but was not completely suppressed.

## 4. Discussion

### 4.1. Involvement of FGF Signaling in the Induction of Cellular Sources of Growth and Regeneration

**Figure 10:**
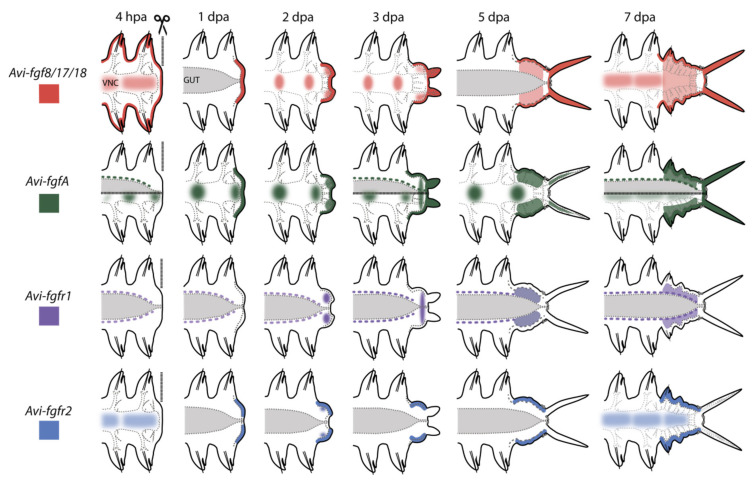
Schematic illustration of gene expression patterns in *A. virens* during posterior regeneration. Anterior is to the left, vertical line at 4 hpa indicates amputation site, the gut is shown in gray, ventral nerve cord (VNC) outlined in gray dotted line. Ectodermal expression is shown in darker lines; mesodermal expression is lighter. Expression pattern of each gene is demonstrated in its own color.

Induction of the proper cellular sources is crucial for successful regeneration. In nereid regeneration, the cells required for this process appear due to dedifferentiation response in the segment abutting the amputation site [4,5,48]. Dedifferentiated cells, rather than stem cells, are considered the source of the blastema in most polychaetes [49,50,51]. It has been experimentally shown that innervation and wound healing are crucial for blastema formation and growth [52,53,54]. However, the molecular inducers of the blastemal precursor cells in annelids have not been identified until now. Our data suggest that FGF is essential for the blastema initiation and the following regenerative process in *A. virens*. In particular, we found an almost immediate (within several hours) response to amputation by expression of FGF genes adjacent to the wound (Figure 3 and Figure 10). Pharmacological blockade starting at this time point leads to the absolute suppression of regeneration.

Expression domains at the 4 hpa stage were noted at the surface of the stump (*Avi-fgf8/17/18*), in the nervous system (for *Avi-fgf8/17/18*, *Avi-fgfA*, *Avi-fgfr2*), in mesodermal cells located around the gut (for *Avi-fgfA*, *Avi-fgfr1*), and at the 1 dpa stage in the wound epithelium (for *Avi-fgf8/17/18*, *Avi-fgfA*, *Avi-fgfr2*). From the stage of 2 dpa, in addition to the domains mentioned above, FGF ligands are expressed in various domains of the blastemal masses and in stripes of the epidermis on the ventral and the lateral sides. Upon 3 dpa, the receptors demonstrate an exclusively mesodermal (*Avi-fgfr1*) or ectodermal (*Avi-fgfr2*) tissue-specific expression. The tissue-specificity of the FGFRs expression in *A. virens* suggests that almost all tissues involved in epimorphic regeneration are potentially competent to FGF. A restricted expression of distinct genes to ectodermal and mesodermal tissues implies that FGF signaling may mediate the interaction of cells derived from these germ layers.

Mutual epidermal-mesodermal interactions are essential during vertebrate limb regeneration, although the role of wound-derived FGFs in the dedifferentiation process is unclear [55]. However, the conserved FGF role in blastema formation is undeniable. FGFs are believed to be nerve-induced factors that can independently promote regeneration without innervation [56]. In urodele tail regeneration, the spinal cord or exogenous FGFs and BMP can induce an ectopic regenerative response in a way similar to the accessory limb model [33]. An extensive activation of the expression of both ligands in the VNC of *A. virens* indicates the possibility of their propagation through neurites to various tissues of the segment.

An early transcriptional response from the epidermis and numerous internal cells in *A. virens* suggests that these structures also act as an FGF source, promoting the recruitment of FGFR-positive mesodermal and ectodermal cells, triggering proliferation and blastema formation. This assumption is supported by (1) the subordination of the FGF expression upregulation and the following onset of an extensive EdU labeling at 2 dpa (Figure 7A), and (2) the inhibition of FGF signaling starting immediately from the moment of amputation (Figure 6). The wound healing is not disturbed under this condition, but neither epithelial nor underlying cells start to divide, which is obvious by the absence of EdU incorporation and tissue outgrowth. According to the vertebrate regeneration models, FGF molecules (and several other growth factors) act as a regulator of the exit from the dormant G0 state into and progression through the G1 phase [57]. Probably, FGF signaling in *A. virens* has a similar effect on differentiated tissues. What controls cell proliferation in the advanced regenerative bud remains to be determined since FGF inhibition at stages after 2 dpa does not completely suppress EdU incorporation.

Our results suggest that FGF signals are possible candidates for reprogramming cell fates in the annelid regeneration. We interpret the mesodermal signal of *Avi-fgfA* and *Avi-fgfr1* in the old segments as a sign that myoepithelial cells might undergo FGF-dependent dedifferentiation processes to populate the wound area and give rise to the blastema. Activation of the germline/multipotency program (GMP) genes [4] in the wound epithelium and just below it confirms this scenario. Indeed, FGFs have important roles in cell survival, migration and proliferation in many developmental contexts. FGF signaling has been shown to promote cell motility and chemotaxis in chick and *Drosophila* embryos [19,58]. FGF-induced dedifferentiation of myocytes and pigment epithelial cells is observed in the regeneration of different elements in the anamniote eye, such as extraocular muscle, lens and retina [10]. Like *A. virens*, the suppression of FGFR or MAPK pathway in an injured zebrafish impairs proliferation, which is a consequence of disturbed reprogramming and dedifferentiation via muscle-to-mesenchyme transition in the zebrafish regeneration [59].

Considering a profound similarity of the FGF requirement for initiation of the whole regenerative processes in the protostome representative (*A. virens*) and vertebrates, we assume that the role of FGFs in cell induction and proliferation may predate the origin of bilaterians. It is tempting to analyze the conservation of the FGF upstream and downstream regulatory circuits in invertebrate models. First of all, we intend to verify whether a local signaling center is formed in the annelid wound epithelium, which stimulates dedifferentiation and recruitment of blastemal cell precursors.

### 4.2. Putative Roles of FGFs in Axial Patterning and Further Regenerative Bud Maturation

Our results demonstrate a high-intensity of expression of FGF components in the ectoderm and the nervous system of the youngest newly formed segments at the late stages of regeneration. This can be considered as a sign of FGFs participating in axis elongation and patterning in *A. virens*, similarly to the dose-dependent FGFs role in vertebrate posterior development [21]. Expression of *A. virens* FGF components in the nervous system near the amputation plane at early stages may represent positional shift (i.e., posteriorization). This hypothesis is supported by the expression profile of some Hox genes, such as *Lox5*, *Lox2* and *Post2* in *A. virens*, which participate in the restoration of the anterior-posterior axis upon amputation [60]. Analyzing regenerative processes in annelids, we called these repatterning events “molecular morphallaxis” [2] and considered them as crucial for the initiation of epimorphic morphogenesis. Expression of the GMP gene *pl10* is also activated de novo at 1 dpa in the posterior-most VNC ganglia [4], which coincides with FGF activation and indicates a change in the regulatory state of the posterior tissues.

Undifferentiated condition is well documented for the wounded area and early regenerative bud of nereids [4,5,48,61]. The observed expression of *A. virens* FGF components in both blastemal and epidermal cells at the 2 dpa stage correlates with the onset of active proliferation in these tissues (Figure 3 and Figure 7). Several GMP marker genes, such as *vasa*, *pl10*, *piwi*, which presumably maintain a multipotent and undifferentiated state of regenerative bud cells in *A. virens*, have an extensive expression in FGF-competent tissues starting from 2 dpa [4]. These facts suggest that FGFs in *A. virens* may act to prevent or slow down premature cell differentiation during blastema growth and patterning (at 2–3 dpa). Moreover, inhibition effects on cell proliferation and morphogenesis are much more prominent at earlier stages when incubation occurs from 2 dpa. Comparing four dpa (Figure 8B,C) and six dpa (Figure 9B,C) regenerates deprived of FGF, we note that after four dpa, the inhibitory effect can be mostly overcome. Interestingly, the inhibitors rarely have any influence on pygidial cirri development. In line with that, these sensory organs demonstrate expressing FGF ligands but not the receptors. Thus, we suppose that in newly formed cirri, FGFs may not control proliferation but act as attractants to the nerves growing into them.

As regeneration proceeds, the bud undergoes consecutive segmentation. Its mechanisms in annelids, as well as its homology in diverse bilaterians, are not fully understood. Though a prevailing hypothesis postulates an independent evolution of segmentation in annelids, arthropods, and chordates [62], significant similarity is recovered in some distantly related species [63]. Segmentation of the body axis proceeds simultaneously with its elongation in both annelid juveniles and vertebrate embryos. Vertebrate FGFs have multiple roles in establishing such borders as the midbrain–hindbrain boundary and the determination front in the paraxial mesoderm [21]. In brachiopod development, *fgf8/17/18* expression does not correlate with ectodermal boundaries of the segments but is associated mostly with metameric chetal sacs [39]. However, our inhibitor experiments apparently influence the segmentation of the regenerative bud if the FGF suppression begins before the blastema patterning at 2–3 dpa (Figure 8 and Figure 9). As a result, we observed fewer segmental boundaries and other metameric structures, whose development was significantly delayed, especially in SU5402. The functional effects correlate with the expression of *Avi-fgfr2*, which has a more prominent signal at segmental borders at 5 and 7 dpa (Figure 3X). At the same stages, *Avi-fgfA* also demonstrates a periodically varying expression intensity with a maximum in the middle of the segment length (Figure 3K,L). Noteworthy, both FGF receptors have no expression in differentiating pygidium, a non-segmented structure. In further studies of FGF in segmented animals, special attention should be paid to its possible involvement in forming boundaries and the patterning of metameres.

### 4.3. Molecular and Functional Evolution of the FGF signaling

FGF molecules activate a fairly ancient signaling pathway [13,15,19]. According to genomic data, the FGF domain and/or its putative predecessor (FGF-like) is found in the predicted genes of choanoflagellates and most metazoans, including sponges ctenophores, cnidarians, and bilaterians. However, only for the representatives of the latter two taxa, the presence of molecules with all the canonical features of FGF ligands and receptors was confirmed. Thus, the signaling pathway mediated by secreted bona fide FGFs has originated in the cnidarian/bilaterian ancestor [9,13,64,65]. Bioinformatic analysis showed a great structural diversity of FGF components in this lineage. Cnidarians possess well-supported FGF8/17/18 members and numerous diverged FGF genes with unclear phylogenetic affiliation [9,64,66]. The same is true of the protostomian repertoire, which in addition acquired representatives of FGF9/16/20 and probably FGF1/2 subfamilies [15,16]. A complete loss of FGF ligands has been documented in certain annelid lineages [41].

The results presented in this study improve our understanding of FGF evolution in protostomes by extending the sampling owing to newly identified and recently described candidate sequences. In our phylogenetic analysis, one of the *A. virens* ligand sequences (named *Avi-fgf8/17/18*) was undoubtedly recovered within FGF8/17/18. This subfamily also includes the ligands found in cnidarians and numerous bilaterians, including the trochozoans *P. dumerilii*, *C. teleta*, *L. gigantea*, lophophorates [40], ecdysozoans and deuterostomes [13,14,15,16]. This broad distribution confirms that FGF8/17/18 is one of the earliest diverged subfamilies. Moreover, FGF8/17/18 genes have a rather conservative role in the early development of bilaterian animals [18,40,67].

Insufficient phylogenetic signal was recovered for FGFs of several distantly related metazoans, including deuterostomes [12,15,26,27]. For *Avi-fgfA*, we were also unable to find well-supported orthology. Due to the low identity percentage between these sister genes of the two nereid species and the lack of other clustered members, they are likely to be fast-evolving sequences rather than representatives of a new subfamily. Since the only described spiralian FGFs outside FGF8/17/18 are members of FGF9/16/20 [15,40], *Avi-fgfA* might belong to either of these subfamilies. Comparative genomics and synteny analysis will help to resolve this issue.

Sequences of FGFR-type tyrosine kinases were identified in sponges (but there are no associated extracellular FGF-binding Ig-like domains), while proper FGFRs are present in all branches of Eumetazoa. Here FGFR paralogs number varies from one (in *C. elegans*, *Tribolium castaneum*, *Strongylocentrotus purpuratus*, *Ciona intestinalis*) to four (in mammals) [46,68]. For *A. virens*, two FGFR paralogs were found, clustering together with the sequences of other spiralians, and differing in the number of Ig-like domains. The number of these domains can differ both in one species and in different clades [68]. The existence of a different number of paralogous sequences in genomes can be the result of lineage-specific duplications, loss or horizontal gene transfer. Probably, similar events occurred independently in different branches of Bilateria and in annelids, for which both common and unique homologs genes of FGF components can be found. Specifically, on the FGFR tree, the sequences of *A. virens*, *P. dumerilii*, and *C. teleta* containing two Ig-like domains tend to segregate from those with three Ig-like domains. It may indicate a duplication event in an annelid or spiralian common ancestor.

When comparing FGF activity in different animals, one can see great diversity and a few conservative features. In *Nematostella vectensis*, 15 ligand-encoding sequences were found, but only 5 of them have been described. For this animal, the involvement of FGF signaling in gastrulation, neurogenesis, and forming the apical organ has been shown, with co-expression of ligands and receptors either in the same place or in very closely situated domains [64,66]. In bilaterian animals, the corresponding expression domains appear in different germ layers, the mesoderm and ectoderm [18]. For early embryos of deuterostome invertebrates, receptor expression domains are confined to the mesoderm and ligands to the ectoderm [12,27,67,69]. The role of FGFs in the induction of the mesoderm adjacent to their source was convincingly demonstrated in numerous bilaterian lineages [18,40,67,70,71]. Overall similar characterization of the FGF function is relevant for insects at the stages of germ-band elongation [72]. In line with that, the MAP-kinase branch of the FGF pathway in *A. virens* also participates in mesoderm development [47,73].

Both ecto- and mesodermal domains are also found in the expression patterns of FGF components in regenerating *A. virens*. However, the presence of two paralogs among the ligands and receptors with different expressional tissue specificity does not allow us to determine the trajectory of intercellular communication with confidence. Our data indicate the fundamental possibility of the mutual influence of internal and superficial tissues, but the autocrine nature of the signaling cannot be ruled out either. Apparently, distinct sets of components and a complex, unique pattern of FGF expression in different taxa reflect a very high evolutionary lability of this system.

## Data Availability

mRNA sequences of *Avi-fgf8/17/18*, *Avi-fgfA*, *Avi-fgfr1* and *Avi-fgfr2* are deposited in GenBank with the accession numbers MZ234756-MZ234759.

## Supplementary Materials

The following are available online at https://www.mdpi.com/article/10.3390/genes12060788/s1, Figure S1: Transverse histological sections after in situ hybridization, which demonstrates expression of the *Avi-fgfr1* at 3 dpa stage in coelomic walls (arrows). A represents more anterior region, than B. Video S2–S5: Stacks of individual images, which demonstrate different focal planes of 3 dpa regenerates. An expression of *Avi-fgf8/17/18* (S2), *Avi-fgfA* (S3), *Avi-fgfr1* (S4), *Avi-fgfr2* (S5) in *A. virens*. Red arrows indicate expression signal in the epidermis of the regenerative bud; green arrows, expression signal in the mesoderm-derived blastema; yellow arrows, expression signal in the mesodermal cells around the gut; black arrows, expression in the ventral cord cells. Asterisks mark non-specific staining. Data S6: Alignment of the FGF ligand core sequence. Data S7: Alignment of the FGFR RTK domain.
