# Supplementary figures and images for: Structural and Functional Characterization of the FGF Signaling Pathway in Regeneration of the Polychaete Worm Alitta virens (Annelida, Errantia)
Source: Genes (Basel). 2021 May 21;12(6):788. doi: 10.3390/genes12060788 (PMC8224027; doi:10.3390/genes12060788)

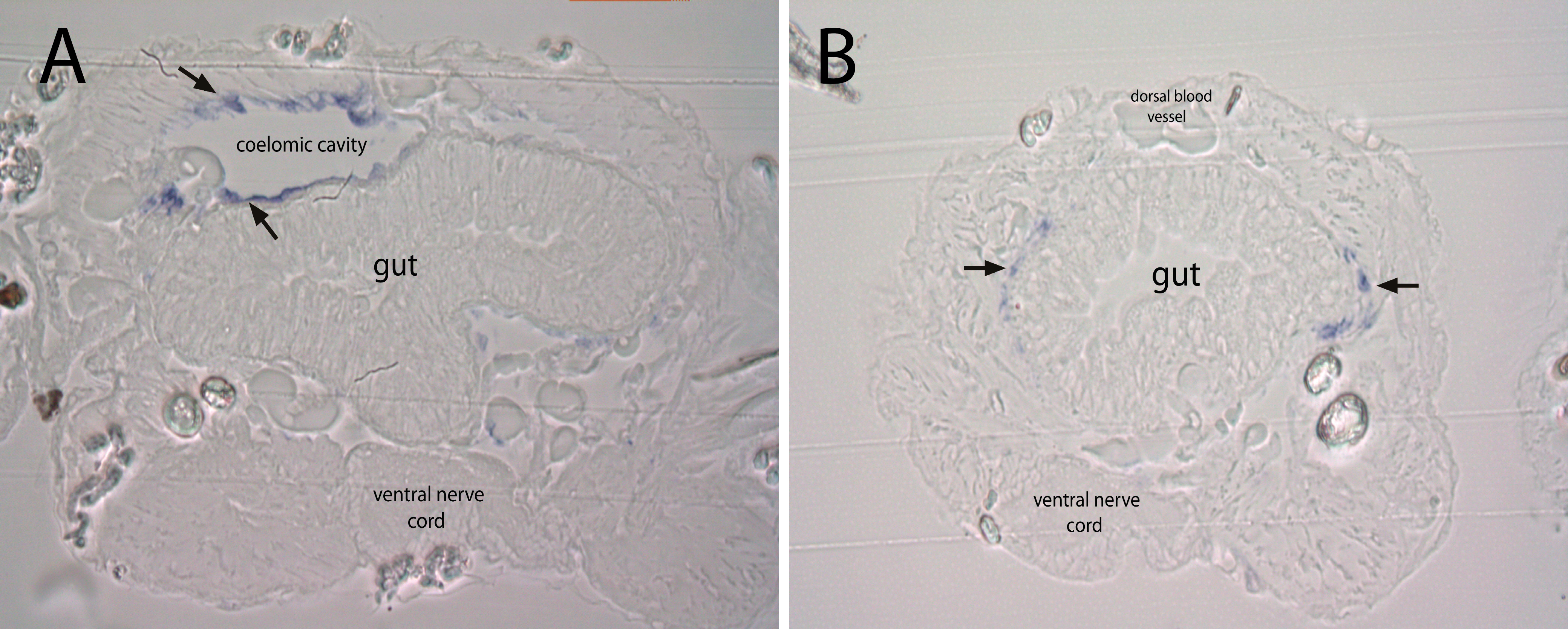

Supplement: Supplementary file 1 [file genes-12-00788-s001.zip › S1.png]
